# Effect of laparoscopic splenectomy in patients with Hepatitis C and cirrhosis carrying IL28B minor genotype

**DOI:** 10.1186/1471-230X-12-158

**Published:** 2012-11-12

**Authors:** Takashi Motomura, Ken Shirabe, Norihiro Furusyo, Tomoharu Yoshizumi, Toru Ikegami, Yuji Soejima, Tomohiko Akahoshi, Morimasa Tomikawa, Takasuke Fukuhara, Jun Hayashi, Yoshihiko Maehara

**Affiliations:** 1 Department of Surgery and Science, Graduate School of Medical Sciences, Kyushu University, Fukuoka, 812-8582, Japan; 2 Department of General Internal Medicine, Graduate School of Medical Sciences, Kyushu University, Fukuoka, Japan; 3 Department of Molecular Virology, Research Institute for Microbial Diseases, Osaka University, Osaka, Japan

**Keywords:** IL28B, ITPA, Splenectomy, Liver cirrhosis

## Abstract

**Background:**

IL28B and ITPA genetic variants are associated with the outcome of pegylated-interferon and ribavirin (PEG-IFN/RBV) therapy. However, the significance of these genetic variants in cirrhotic patients following splenectomy has not been determined.

**Methods:**

Thirty-seven patients with HCV-induced cirrhosis who underwent laparoscopic splenectomy (Spx group) and 90 who did not (non-Spx group) were genotyped for IL28B and ITPA. The outcome or adverse effects were compared in each group. Interferon-stimulated gene 15 (ISG15) and protein kinase R expression in the spleen was measured using total RNA extracted from exenterate spleen.

**Results:**

Sustained virological response (SVR) rate was higher in patients carrying IL28B major genotype following splenectomy (50% vs 27.3%) and in patients carrying minor genotype in the Spx group compared to non-Spx group (27.3% vs 3.6%, *P* < 0.05). Pretreatment splenic ISG expression was higher in patients carrying IL28B major. There was no difference in progression of anemia or thrombocytopenia between patients carrying each ITPA genotype in the Spx group. Although splenectomy did not increase hemoglobin (Hb) level, Hb decline tended to be greater in the non-Spx group. In contrast, splenectomy significantly increased platelet count (61.1 × 10^3^/μl vs 168.7 × 10^3^/μl, *P* < 0.01), which was maintained during the course of PEG-IFN/RBV therapy.

**Conclusions:**

IL28B genetic variants correlated with response to PEG-IFN/RBV following splenectomy. Splenectomy improved SVR rate among patients carrying IL28B minor genotype and protected against anemia and thrombocytopenia during the course of PEG-IFN/RBV therapy regardless of ITPA genotype.

## Background

Hepatitis C virus (HCV) chronically infects over 170 million people worldwide, with 3–4 million individuals newly infected each year. Liver cirrhosis may progress in 30% of chronic hepatitis C patients, and even hepatocellular carcinomas develop in 7–8% of cirrhotic patients annually [1,2]. Although the current standard therapy for HCV is combination of pegylated-interferon plus ribavirin (PEG-IFN/RBV), sustained virological response (SVR), defined as negativity for HCV RNA for 24 weeks after cessation of therapy, can be achieved for only 50% of chronic hepatitis patients, and for <30% of cirrhotic patients [3,4]. In addition to low efficacy, many adverse effects, especially cytopenia, are extremely detrimental to cirrhotic patients.

Splenectomy is recommended for cirrhotic patients with thrombocytopenia to enable them to receive PEG-IFN/RBV therapy safely [5]. Although patients with liver cirrhosis have a high risk of hemorrhage due to dilated collateral vessels, splenomegaly, and poor liver function, we have demonstrated the safety and feasibility of laparoscopic splenectomy (LS) since its first report in 1992 [6,7]. In particular, it could be very effective for cirrhotic patients who have failed induction of PEG-IFN/RBV therapy due to low platelet counts [8].

A genome-wide association study (GWAS) has revealed two striking single nucleotide polymorphisms (SNPs), IL28B and ITPA, which are correlated with the outcome or adverse effects of PEG-IFN/RBV therapy [9-11]. A lower VR rate has been shown in patients carrying the minor genotype (TG or GG) at rs8099917 near the IL28B locus, and treatment-induced hemolytic anemia has been reported more often in patients carrying the major genotype (CC) at rs1127354 in the ITPA gene. In addition, ITPA genetic variants have also been reported recently to correlate with platelet reduction during PEG-IFN/RBV therapy [12].

However, the significance of these two SNPs among patients who have undergone LS has yet to be determined. For more than a little patients could not achieved virological response or would failed the incessancy of PEG-IFN/RBV even after splenectomy, it would be very beneficial to predict the outcomes of anti-HCV therapy following splenectomy. In the current study, we demonstrated the association of IL28B and ITPA SNPs with the outcome of PEG-IFN/RBV therapy following LS.

## Methods

### Patients

From August 2004 to March 2009, 117 consecutive type C cirrhotic patients underwent LS at our institute for the induction of PEG-IFN/RBV therapy, and DNA was available for genotyping from 37 of these. They were compared to 90 cirrhotic patients who did not undergo splenectomy before induction of PEG-IFN/RBV therapy in the same period at the Department of General Internal Medicine, Kyushu University. Patients in the control group were selected on account of their platelet count <10^5^/ml or by METAVIR fibrosis stage F4 [13]. Therefore, 127 patients were enrolled in the current study, which was approved by the Ethics Committee of Kyushu University.

### DNA extraction and genotyping

Genomic DNA was extracted from the patients’ spleen tissues obtained at operation, or from peripheral blood mononuclear cells in the control group. IL28B genetic polymorphism (rs8099917) and ITPA genetic polymorphism (rs1127354) were genotyped using StepOnePlus™ real-time PCR system (Applied Biosystems, Carlsbad, CA, USA).

### Definition of outcomes of PEG-IFN/RBV therapy

VR was defined as a lack of HCV RNA in response to the treatment regimen, regardless of whether a relapse occurred when treatment was terminated. SVR was defined as undetectable HCV-RNA 24 weeks after the end of the therapy. Patients who had achieved VR but had showed relapse were defined as end of treatment response (ETR)-relapse. Hemoglobin (Hb) level or platelet count was assessed before splenectomy, at induction of therapy, and every 4 weeks during the course of PEG-IFN/RBV therapy.

### RNA extraction and reverse transcriptase polymerase chain reaction (RT-PCR)

Total RNA was extracted from exenterate spleen specimens using ISOGEN (Nippon Gene, Tokyo, Japan) and reverse transcription was performed using SuperScriptIII (Invitrogen, Carlsbad, CA, USA). Quantitative PCR was performed using SYBR® Green assay (Applied Biosystems) on the LightCycler®480 Real-Time PCR system (Roche Applied Sciences, Indianapolis, IN, USA). Specific primers for ISG15 were as follows: 5^′^-agcgaa ctcatctttg-3^′^ for sense primer 5^′^-cagctctgacaccgacatgga-3^′^ for antisense primer. Specific primers for protein kinase R (PKR) were 5^′^-acgtgtgagtcccaaagcaac-3^′^ for sense and 5^′^-ctgagaccattcataagcaacg-3^′^ for antisense. β-Actin expression was used for endogenous control with 5^′^-ct ggcaccacac cttctacaatg-3^′^ for sense primer and 5^′^-ggcgtacagggatagcacagc-3^′^ for antisense primer.

### Statistical analysis

All data were analyzed using JMP® statistical software (SAS Institute, Cary, NC, USA). A χ^2^ test was performed for qualitative variables and a Wilcoxon test was performed for quantitative variables.

## Results

### Patients’ characteristics

Characteristics of patients who underwent splenectomy (Spx) and those who did not (non-Spx) are shown in Table 1. Although patients in the non-Spx group were selected on account of their platelet count <10^5^/μl, count before surgery in the Spx group was lower than that in the non-Spx group (6.1 × 10^4^/μl vs 8.7 × 10^4^/μl, respectively, *P* < 0.0001), as well as their Hb level (12.5 g/dl vs 13.2 g/dl, respectively, *P* = 0.03). Both alanine aminotransferase and γ-glutamyl transpeptidase levels were significantly higher in the non-Spx group (55 IU/l vs 91 IU/l, *P* = .001, 45 IU/l vs 60 IU/l, *P* = 0.02, respectively), but albumin level was higher in the non-Spx group (3.3 mg/dl vs 3.9 mg/dl, *P* < 0.0001). The progression of cirrhosis was considered to be more severe in the Spx group. Neither pretreatment viral load nor the frequency of HCV genotype differed among these groups. The allele frequency of both IL28B (rs8099917) and ITPA (rs1127354) genetic polymorphisms was similar in these groups.

**Table 1:** Data Among HCV-positive patients who underwent splenectomy (Spx group) and who did not (non-Spx group)

	**Spx group (n=37)**	**Non-Spx group (n=90)**	**P value**
Age (y), mean ± SD	58 ±1	61 ± 1	n.s
Sex (male / female), n	19 / 18	44 / 46	n.s
Body Mass Index (kg/m^2^), mean ± SD	24.9 ± 0.5	22.7 ± 0.5	0.002
Hemoglobin level (g/dl), mean ± SD	12.5 ± 0.3	13.2 ± 0.2	0.03
Platelet count (x10^4^/μl), mean ± SD	5.9 ± 0.2	8.7 ± 0.2	<0.0001
Alanine aminotransferase level (IU/L), mean ± SD	55 ± 10	91 ± 7.9	0.001
Gamma-glutamyle transpeptidase (IU/L), mean ± SD	45 ± 7.4	60 ± 5.1	0.02
Albumin level (g/dl), mean ± SD	3.3 ± 0.07	3.9 ± 0.06	<0.0001
Pretreatment viral load (logIU/mL), mean ± SD	5.9 ± 0.1	6.2 ± 0.1	n.s
HCV genotype (1/2), n	32 / 5	69 / 21	n.s
IL28B genotype (major/minor), n	26 / 11	62 / 28	n.s
ITPA genotype (major/minor), n	26 / 11	61 / 29	n.s

HCV; hepatitis C virus, IL28B; interleukin-28B, ITPA; inosine triphosphate pyrophosphatase, n.s ; not significant.

Spx; splenectomy.

### IL28B Genotype and therapeutic effect of PEG-IFN/RBV following splenectomy

**Figure 1:**
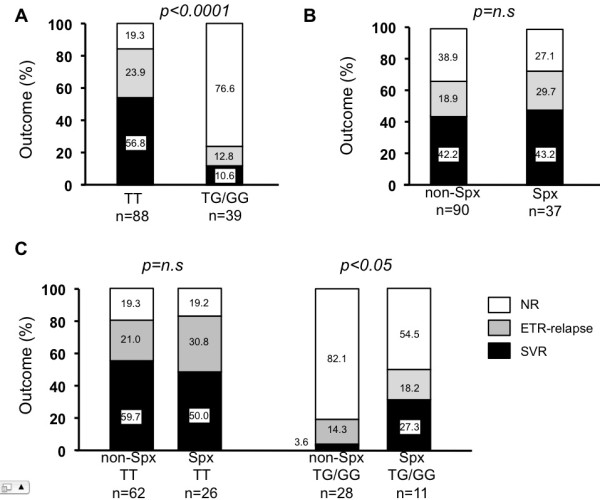
**IL28B genetic polymorphism and response to PEG-IFN/RBV (black bar: SVR, grey bar: ETR-relapse, white bar: NR).** Each number represents the rate in percentile). (**A**) IL28B genetic polymorphism and response rate among patients with HCV-induced cirrhosis. (**B**) Response rate between Spx and non-Spx groups. (**C**) IL28B genetic polymorphism and each response rate in the Spx and non-Spx groups.

Fifty-four of 127 patients achieved a SVR (42.5%). Patients carrying IL28B the major genotype (TT allele at rs8099917, n = 88) showed a significantly higher SVR rate compared to those carrying the minor genotype (TG or GG, n = 39) (56.8% vs 10.3%, *P* < 0.0001, Figure 1A). To clarify the effect of splenectomy on the outcome of PEG-IFN/RBV therapy, SVR rate was compared between the Spx and non-Spx groups. Although the ETR-relapse rate in the Spx group seemed higher than in the non-Spx group (27.0% vs 18.9%), there was no significant difference in SVR rate between the two groups (43.2% vs 42.3%, *P* =0.91, Figure 1B).

SVR rate among each IL28B genotype in the Spx and non-Spx groups was compared. As shown in Figure 1C, IL28B genetic variants were significantly associated with SVR rate in the non-Spx group (58.9% vs 4.1%, *P* < 0.0001). SVR rate in the Spx group was not different between IL28B genotype (50% vs 27.3%, *P=*0.09), probably because of small number, but VR rate (SVR + ETR-relapse) of Spx group was higher in IL28B major group compared to those in minor group (80.8% vs 45.5%, *P* <0 .05). Interestingly, patients carrying the minor genotype in the Spx group showed a significantly higher SVR rate than those in the non-Spx group (27.3% vs 3.6%, *P* < 0.05), whereas there was no difference in SVR rate among patients carrying the major genotype between the SPx and non-Spx groups (50.0% vs 59.7%, *P* = 0.40).

It is well-known that IFN response is correlated with HCV genotype. Therefore, we made the same analysis on 100 patients infected with only genotype 1 HCV. The similar results were obtained, but no statistical difference in SVR rate among IL28B minor allele carriers between Spx- and non-Spx-group, probably because of small number (20.0% vs 3.7%, data not shown).

Next, we checked the prognostic factors for achieving SVR among IL28B minor allele carriers. As shown in Table 2, splenectomy was the only significant factors for SVR with odds ratio of 10.1 (p<0.05).

**Table 2:** Comparison of Data Among IL28B minor allele carriers who achieved SVR or not

	**SVR group (n=4)**	**Non-SVR group (n=35)**	**Odds ratio**	**P value**	**95% CI**
Age ≥60 (yes / no), n	1 / 3	23 / 12	0.17	n.s	0.01 – 1.85
Sex (male / female), n	2 / 2	19 / 16	0.84	n.s	0.11 – 6.67
Hemoglobin level (g/dl), mean ± SD	13.2 ± 0.8	12.9 ± 0.3	-	n.s	-
Platelet count (x10^4^/μl), mean ± SD	7.9 ± 0.9	8.0 ± 0.3	-	n.s	-
Pretreatment viral load (logIU/mL), mean ± SD	6.5 ± 0.4	6.3 ± 0.1	-	n.s	-
HCV genotype (1/2), n	3 / 1	34 / 1	11.3	0.06	0.56 - 230
splenectomy (yes / no), n	3 / 1	8 / 27	10.1	<0.05	0.92-112
ITPA genotype (major/minor), n	3 / 1	26 / 9	1	n.s	0.09 – 11.2

CI; confidence interval, HCV; hepatitis C virus, IL28B; interleukin-28B, ITPA; inosine triphosphate pyrophosphatase, n.s ; not significant, Spx; splenectomy.

### ITPA genotype and PEG-IFN/RBV-induced anemia following splenectomy

**Figure 2:**
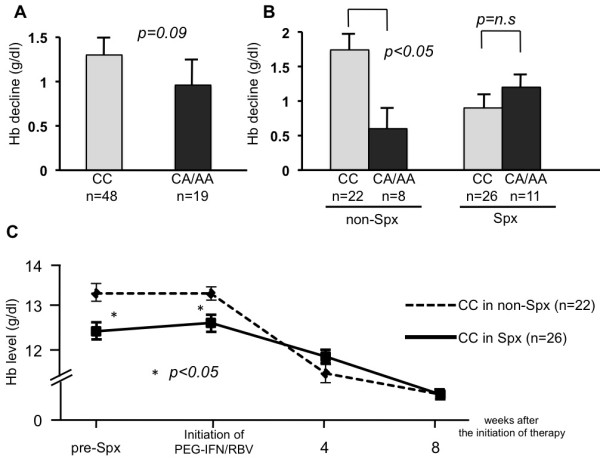
**ITPA genetic polymorphism and PEG-IFN/RBV-induced anemia.** (**A**) ITPA genetic polymorphism and Hb decline at 4 weeks after initiation of therapy among patients with HCV-induced cirrhosis. (**B**) ITPA genetic polymorphism and treatment-induced anemia in Spx and non-Spx groups. (**C**) The changes of Hb levels among ITPA major carriers in the comparison of Spx and non-Spx groups.

Hb level and platelet count at each time point were available for 30 patients in the non-Spx group. Although Hb decline at 4 weeks after the initiation of PEG-IFN/RBV tended to be greater in patients carrying the CC allele than CA/AA allele (1.34 g/dl vs 0.96 g/dl, *P* = 0.09, Figure 2A), there was no significant difference among them. However, patients carrying the CC allele in the non-Spx group were more susceptible to anemia than those carrying the CA/AA allele (Hb decline of 1.74 g/dl vs 0.60 g/dl, *P* < 0.05), whereas there was no difference in Hb decline between patients carrying each genotype in the Spx group (0.96 g/dl vs 1.28 g/dl, *P* = 0.89, Figure 2B).

In addition, progression of anemia among patients carrying the CC allele was compared in each group (Figure 2C). Before splenectomy, Hb level in patients in the Spx group was lower than in the non-Spx group (13.2 g/dl vs 12.3 g/dl, *P* < 0.05). In the Spx group, Hb level did not change after splenectomy (12.3 g/dl vs 12.5 g/dl, *P* = 0.50) and was still lower than that in the non-Spx group (13.2 g/dl vs 12.5 g/dl, *P* < 0.05). However, Hb levels at 4 and 8 weeks after the initiation of PEG-IFN/RBV were similar in each group (11.3 g/dl vs 11.7 g/dl, *P* = 0.55, 10.8 g/dl vs 10.8 g/dl, *P* = 0.89, respectively).

### ITPA genotype and platelet reduction during PEG-IFN/RBV following splenectomy

**Figure 3:**
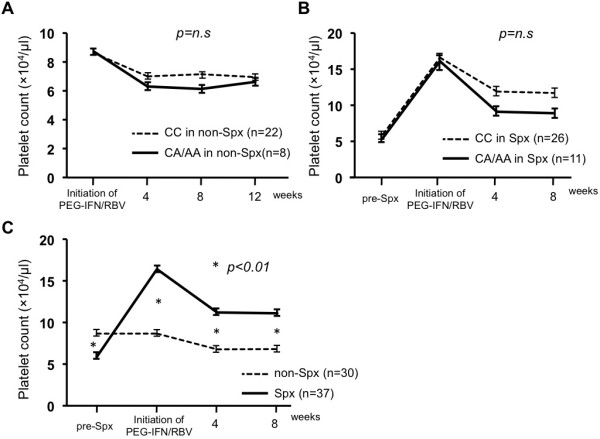
**ITPA genetic polymorphism and thrombocytopenia.** (**A**) The progression of thrombocytopenia between CC and CA/AA allele carriers in non-Spx group. (**B**) The progression of thrombocytopenia between CC and CA/AA allele carriers in Spx group. (**C**) The changes of platelet count was compared between Spx and non-Spx groups.

Progression of thrombocytopenia in patients carrying each ITPA genotype was compared in each group. There was no difference in platelet count before PEG-IFN/RBV between patients carrying the CC allele or CA/AA allele (86.2 × 10^3^/μl vs 86.9 × 10^3^/μl, *P* = 0.86). Progression of thrombocytopenia tended to be worse in patients carrying the CA/AA allele, as reported previously, but there was no significant difference in platelet count at 4, 8 and 12 weeks after initiation of PEG-IFN/RBV (69.9 × 10^3^/μl vs 63.2 × 10^3^/μl; *P* = 0.26, 71.5 × 10^3^/μl vs 61.3 × 10^3^/μl; *P* = 0.23, 69.5 × 10^3^/μl vs 66.2 × 10^3^/μl, *P* = 0.28, respectively, Figure 3A).

Compared with pre- and postoperative platelet count, although splenectomy improved thrombocytopenia in whole splenectomy patients (61.1 × 10^3^/μl vs 168.7 × 10^3^/μl, *P* < 0.01, data not shown), there was no difference in pre- or postoperative platelet count between patients carrying the CC and CA/AA allele (60.4 × 10^3^/μl vs 55.4 × 10^3^/μl; *P* = 0.68, 165.8 × 10^3^/μl vs 160.9 × 10^3^/μl, *P* = 0.86, respectively). As shown in Figure 3B, however, platelet counts in splenectomy patients carrying the CA/AA allele tended to be lower than in those carrying the CC allele at 4 and 8 weeks after initiation of PEG-IFN/RBV (118.8 × 10^3^/μl vs 91.6 × 10^3^/μl; *P* = 0.12, 117.4 × 10^3^/μl vs 89.3 × 10^3^/μl, *P* = 0.14, respectively) without any significant difference.

To clarify the efficacy of splenectomy in thrombocytopenia, platelet counts were compared between the Spx and non-Spx groups at each time point (Figure 3C). The patients in the non-Spx group were selected on account of their platelet count <10^5^/μl, but those whose platelet count was <50–60 × 10^3^/μl were seldom induced with PEG-IFN/RBV, which led to a significant difference in platelet count before splenectomy (86.7 × 10^3^/μl vs 58.9 × 10^3^/μl, *P* < 0.01). However, splenectomy increased the platelet count in the Spx group to overtake that in the non-Spx group (86.7 × 10^3^/μl vs 164.3 × 10^3^/μl, *P* < 0.01), and its dominance was maintained even at 4 and 8 weeks after initiation of PEG-IFN/RBV (67.7 × 10^3^/μl vs 112.2 × 10^3^/μl; *P* < .01, 68.1 × 10^3^/μl vs 111.3 × 10^3^/μl, *P* = 0.01, respectively).

### IL28B Genotype and splenic ISG expression

**Figure 4:**
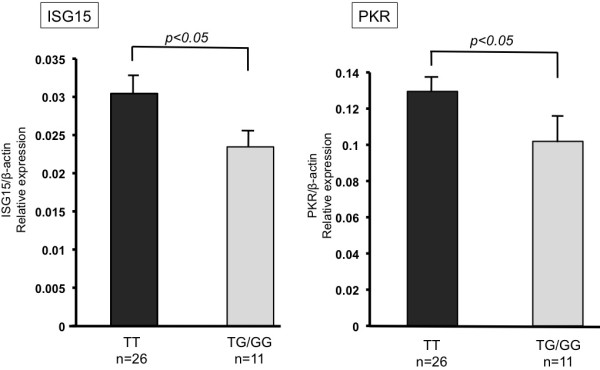
**IL28B genetic polymorphism and splenic ISG expression.** Both splenic ISG15 and PKR mRNA expression was higher in patients carrying the TT allele of IL28B.

The correlation of IL28B and ITPA genetic variants with the outcome of PEG-IFN/RBV therapy following splenectomy was demonstrated. IL28B genetic variant has also been reported to correlate with hepatic ISG expression among chronic hepatitis C patients [14]. Whether IL28B genetic variant also correlated with splenic ISG expression has yet to be determined. Surprisingly, as shown in Figure 4, both ISG15 and PKR expression in the spleen was significantly higher in patients carrying the TT allele than the TG/GG allele, which was opposite to liver tissue as described previously (0.03 vs 0.02; *P* < 0.05, 0.13 vs 0.10; *P* < 0.05, respectively).

## Discussion

Recent developments in genome sequence techniques have enabled GWASs, which have resulted in tailored therapies for various diseases. For patients affected with HCV-related liver diseases, two important genetic polymorphisms, IL28B and ITPA, have been reported recently, which hopefully will lead to appropriate antiviral therapy for each individual.

Although splenectomy is useful for improving liver function in type C cirrhotic patients for the induction of PEG-IFN/RBV therapy, even less invasive laparoscopic surgery carries some risks and has a high cost. The precise prediction for the outcome of PEG-IFN/RBV therapy following LS is urgently needed.

The current study revealed that IL28B genetic variant also correlated with response to PEG-IFN/RBV following LS. Interestingly, compared to cirrhotic patients who had not undergone splenectomy (non-Spx group) and were carrying the TG/GG allele at the IL28B gene, those who underwent splenectomy (Spx group) and carried the TG/GG allele had a significantly higher SVR rate, whereas TT carriers showed no difference between the SPx and non-Spx groups.

One of the mechanisms by which splenectomy improves antiviral response could be the restoration of cytopenia, leading to enhanced treatment tolerance. Although patients carrying the CC allele at the ITPA gene had greater susceptibility to anemia in the non-Spx group, there was no difference between the ITPA genotypes in the Spx group. Hitomi et al [15]. recently clarified that ITPA genetic variant protected anemia by accumulated inosine triphosphates been used for decreased ATP via adnylosuccsinate synthase. In that study, however, the authors reported that erythrocytes with RBV *in vitro* did not show hemolysis by themselves, suggesting that hemolysis occurred in other tissues *in vivo*. In the current study, splenectomy did not improved anemia, which was significantly worse than that in the non-Spx group. Nevertheless, Hb levels during the course of PEG-IFN/RBV therapy were similar in the Spx and non-Spx groups among patients carrying the CC allele. These data suggest that splenectomy does not improve anemia, but protects against RBV-induced hemolysis, especially among patients carrying the ITPA CC allele.

In contrast to anemia, splenectomy itself significantly improved thrombocytopenia, which enabled us to perform PEG-IFN/RBV therapy safely.

The restoration of peripheral blood cells does not explain why SVR was improved by splenectomy in patients carrying the IL28B minor genotype. The current study also revealed that splenic ISG expression was higher in patients carrying the IL28B major genotype, which was opposite to the liver. We consider that this resulted from the difference in constituent cells in each organ. In fact, the liver mainly comprises hepatocytes, whereas the spleen mainly comprises monocytes. In a previous report, IL28 mRNA expression was higher in peripheral blood monocytes of patients carrying the TT allele than those carrying the TG/GG allele [9]. ISG expression was also reported to be higher in liver-infiltrating lymphocytes of responders to PEG-IFN/RBV than those of non-responders [16]. Although it is unclear whether monocytes have similar phenotypes in peripheral blood, liver and spleen, pretreatment ISG expression was higher in monocytes of patients carrying the IL28B major genotypes. Similarly, Chen et al. have shown by immunohistochemistry that ISG15 protein upregulation was pronounced in Kuppfer cells among responders to IFN therapy [17]. Contrary to monocytes, ISGs from liver parenchyma or hepatocytes are known to be greater in number in non-responders to PEG-IFN/RBV [16,18]. These data suggest that pretreatment ISG upregulation in hepatocytes in which HCV replicates induces refractoriness to IFN, whereas monocytes that express little endogenous ISG have some dysfunction for eradication of HCV. Spleens in patients carrying the IL28B minor genotype have some of these dysfunctional monocytes. Therefore, it is possible that splenectomy removes such monocytes and improves the outcome of PEG-IFN/RBV therapy, especially in patients carrying the IL28B minor genotype.

In addition, we have previously demonstrated that the inhibitory signal, programmed-death 1(PD-1), is upregulated in splenic CD4^+^ T cells and they promote peripheral tolerance to IFN [19]. In fact, splenectomy is followed by an increase in cytokine production and a reduction of PD-1 in peripheral blood lymphocytes. Not only PD-1, but also Tim-3, a well-known inhibitory signal, are upregulated in HCV-specific T cells, which are predominantly central memory T cells that are located in lymphatic tissues such as the spleen [20]. These data suggest that splenectomy does improve the immune response to HCV, but confirmation of the correlation of IL28B minor genotype with inhibitory signals in T cells needs further investigation.

## Conclusion

In conclusion, IL28B genetic polymorphism was also correlated with response to PEG-IFN/RBV among cirrhotic patients following splenectomy and splenic ISG expression. Splenectomy improved SVR rate, especially among patients carrying the IL28B minor genotype, protecting against hemolytic anemia or thrombocytopenia, regardless of ITPA genetic variants.

## Abbreviations

GWAS: Genome-wide association study; Hb: Hemoglobin; HCV: Hepatitis C virus; ISG: Interferon-stimulated gene; LS: Laparoscopic splenectomy; PD-1: Programmed death-1; PEG-IFN: Pegylated-interferon; RBV: Ribavirin; RT-PCR: Reverse transcriptase polymerase chain reaction; SNP: Single nucleotide polymorphism; Spx: Splenectomy; SVR: Sustained virological response; ETR: End of treatment response; VR: Virological response.

## Competing interest

The authors of this manuscript declare that there are no competing interests with regards to this work.

## Authors’ contribution

TM designed and performed the research, acquired and analyzed the data, and wrote the paper; KS designed the research and wrote the paper; NF performed the research and acquired the data; TY, TI and YS provided clinical information; TA and MT performed surgery; TF designed the research; and JH and YM edited the paper. All authors read and approved the final manuscript.

## Pre-publication history

The pre-publication history for this paper can be accessed here:

http://www.biomedcentral.com/1471-230X/12/158/prepub

## References

[B1] Global Burden of Hepatitis C Working Group Global burden of disease (GBD) for hepatitis C J Clin Pharmacol 2004 44 20 29 14681338 10.1177/0091270003258669

[B2] Kiyosawa K Umemura T Ichijo T Matsumoto A Yoshizawa K Gad A Hepatocellular carcinoma: recent trends in Japan Gastroenterology 2004 127 S17 S26 10.1053/j.gastro.2004.09.012 15508082

[B3] Hadziyannis SJ Sette H Jr Morgan TR Balan V Diago M Marcellin P Peginterferon-alpha2a and ribavirin combination therapy in chronic hepatitis C: a randomized study of treatment duration and ribavirin dose Ann Intern Med 2004 140 5 346 355 14996676 10.7326/0003-4819-140-5-200403020-00010

[B4] Bruno S Shiffman ML Roberts SK Gane EJ Messinger D Hadziyannis SJ Marcellin P Efficacy and safety of peginterferon alfa-2a (40KD) plus ribavirin in hepatitis C patients with advanced fibrosis and cirrhosis Hepatology 2010 51 2 388 397 10.1002/hep.23340 19918980

[B5] Kumada H Okanoue T Onji M Moriwaki H Izumi N Tanaka E Guidelines for the treatment of chronic hepatitis and cirrhosis due to hepatitis C virus infection for the fiscal year 2008 in Japan Hepatol Res 2010 40 1 8 13 10.1111/j.1872-034X.2009.00634.x 20156296

[B6] Hashizume M Sugimachi K Ueno K Laparoscopic splenectomy with an ultrasonic dissector N Engl J Med 1992 327 6 438 1385643 10.1056/NEJM199208063270621

[B7] Tomikawa M Akahoshi T Sugimachi K Ikeda Y Yoshida K Tanabe Y Kawanaka H Takenaka K Hashizume M Maehara Y Laparoscopic splenectomy may be a superior supportive intervention for cirrhotic patients with hypersplenism J Gastroenterol Hepatol 2010 25 2 397 402 10.1111/j.1440-1746.2009.06031.x 19929930

[B8] Akahoshi T Tomikawa M Kawanaka H Furusyo N Kinjo N Tsutsumi N Laparoscopic splenectomy with IFN therapy in one hundred HCV-cirrhotic patients with hypersplenism and thrombocytopenia J Gastroenterol Hepatol 2012 27 2 286 290 10.1111/j.1440-1746.2011.06870.x 21793908

[B9] Tanaka Y Nishida N Sugiyama M Kurosaki M Matsuura K Sakamoto N Genome-wide association of IL28B with response to pegylated interferon-alpha and ribavirin therapy for chronic hepatitis C Nat Genet 2009 41 10 1105 1109 10.1038/ng.449 19749757

[B10] Fellay J Thompson AJ Ge D Gumbs CE Urban TJ Shianna KV ITPA gene variants protect against anaemia in patients treated for chronic hepatitis C Nature 2010 464 7287 405 408 10.1038/nature08825 20173735

[B11] Ochi H Maekawa T Abe H Hayashida Y Nakano R Kubo M ITPA polymorphism affects ribavirin-induced anaemia and outcomes of therapy–a genome-wide study of Japanese HCV virus patients Gastroenterology 2010 139 4 1190 1197 10.1053/j.gastro.2010.06.071 20637204

[B12] Tanaka Y Kurosaki M Nishida N Sugiyama M Matsuura K Sakamoto N Genome-wide association study identified ITPA/DDRGK1 variants reflecting thrombocytopenia in pegylated interferon and ribavirin therapy for chronic hepatitis C Hum Mol Genet 2011 20 17 3507 3516 10.1093/hmg/ddr249 21659334

[B13] Bedossa P Poynard T An algorithm for the grading of activity in chronic hepatitis C Hepatology 1996 24 2 289 293 10.1002/hep.510240201 8690394

[B14] Honda M Sakai A Yamashita T Nakamoto Y Mizukoshi E Sakai Y Hepatic ISG expression is associated with genetic variation in interleukin 28B and the outcome of IFN therapy for chronic hepatitis C Gastroenterology 2010 139 2 499 509 10.1053/j.gastro.2010.04.049 20434452

[B15] Hitomi Y Cirulli ET Fellay J McHutchison JG Thompson AJ Gumbs CE Inosine triphosphate protects against ribavirin-induced adenosine triphosphate loss by adenylosuccinate synthase function Gastroenterology 2011 140 4 1314 1321 10.1053/j.gastro.2010.12.038 21199653

[B16] Honda M Nakamura M Tateno M Sakai A Shimakami T Shirasaki T Differential interferon signaling in liver lobule and portal area cells under treatment for chronic hepatitis C J Hepatol 2010 53 5 817 826 10.1016/j.jhep.2010.04.036 20739080

[B17] Chen L Borozan I Sun J Guindi M Fischer S Feld J Cell-type specific gene expression signature in liver underlies response to interferon therapy in chronic hepatitis C infection Gastroenterology 2010 138 3 1123 1133 10.1053/j.gastro.2009.10.046 19900446

[B18] Asahina Y Izumi N Hirayama I Tanaka T Sato M Yasui Y Potential relevance of cytoplasmic viral sensors and related regulators involving innate immunity in antiviral response Gastroenterology 2008 134 5 1396 1405 10.1053/j.gastro.2008.02.019 18471516

[B19] Hashimoto N Shimoda S Kawanaka H Tsuneyama K Uehara H Akahoshi T Modulation of CD4+ T cell responses following splenectomy in hepatitis C virus-related liver cirrhosis Clin Exp Immunol 2011 165 2 243 250 10.1111/j.1365-2249.2011.04393.x 21615390 PMC3142649

[B20] McMahan RH Golden-Mason L Nishimura MI McMahon BJ Kemper M Allen TM Tim-3 expression on PD-1+ HCV-specific human CTLs is associated with viral persistence, and its blockade restores hepatocyte-directed in vitro cytotoxicity J Clin Invest 2010 120 12 4546 4557 10.1172/JCI43127 21084749 PMC2994339

